# Utilization of a Deep Learning Algorithm for Automated Segmentation of Median Nerve from Ultrasound Obtained from the Distal Forearm and Wrist

**DOI:** 10.3390/bioengineering12121289

**Published:** 2025-11-24

**Authors:** Amad Qureshi, Kyle Tse, Siddhartha Sikdar, Yonatan Serlin, Atsede Akalu, Tianxia Wu, Katharine Alter, Qi Wei, Tanya Lehky

**Affiliations:** 1 Department of Bioengineering, George Mason University, Fairfax, VA 22030, USA; aquresh@gmu.edu (A.Q.); ssikdar@gmu.edu (S.S.); qwei2@gmu.edu (Q.W.); 2 Electromyography Section, National Institute of Neurological Disorders and Stroke, National Institutes of Health, Bethesda, MD 20892, USA; atsede.akalu@nih.gov (A.A.); lehkyt@ninds.nih.gov (T.L.); 3 Mid-Atlantic Permanente Medical Group, Department of Neurology, Washington, DC 20002, USA; 4 Neurophysiology of Epilepsy Unit, National Institute of Neurological Disorders and Stroke, National Institutes of Health, Bethesda, MD 20892, USA; yonatan.serlin@nih.gov; 5 Clinical Trials Unit, National Institute of Neurological Disorders and Stroke, National Institutes of Health, Bethesda, MD 20892, USA; wuti@mail.nih.gov; 6 Department of Rehabilitation Medicine, Clinical Center, National Institutes of Health, Bethesda, MD 20892, USA; kalter@cc.nih.gov

**Keywords:** artificial intelligence, median nerve, nerve segmentation, ultrasound, U-Net

## Abstract

Carpal tunnel syndrome (CTS) is the most common peripheral nerve entrapment, and ultrasound provides a fast, cost-efficient method for visualizing the median nerve. Reliable cross-sectional area (CSA) measurement remains challenging across imaging sites and varying scan qualities, and prior studies report segmentation Dice scores ranging from 0.76 to 0.93. Improving the robustness of automated segmentation is critical for achieving consistent, site-independent CSA assessment. This study evaluates a four-layer U-Net for automated segmentation and CSA estimation at two clinically relevant sites: the wrist crease and distal forearm. A primary dataset of 500 images per site was used to establish baseline performance. A second dataset of 35 wrist and 26 forearm still images was used to test generalizability, followed by intensity-based augmentations (CLAHE, gamma correction, speckle noise). Baseline models were tested on these new stills, and an augmented model was trained and evaluated on the combined datasets. The baseline models performed well on the first dataset but showed markedly reduced generalization on new still images (forearm IoU/Dice: 0.185/0.254; wrist IoU/Dice: 0.137/0.188). The augmented models improved within-set performance (forearm: 0.944/0.971; wrist: 0.951/0.974) and significantly enhanced generalization to new images (forearm: 0.408/0.533; wrist: 0.705/0.820). The final combined dataset models achieved Dice/IoU scores of 0.94/0.89 (forearm) and 0.96/0.92 (wrist). CSA measurements showed excellent to moderate correlation with manual tracings across all datasets. These findings demonstrate that targeted intensity-based augmentation substantially improves model generalization and enables robust, reproducible, and site-independent median nerve segmentation, supporting scalable ultrasound-based CTS assessment.

## 1. Introduction

Ultrasonography has become an increasingly popular tool in neurology practice due to its portability, lack of ionizing radiation, real-time dynamic imaging of vascular and nerve structures, and cost-effectiveness compared to other imaging modalities, such as MRI or CT [1,2]. Recent advances in high- and ultra-high-frequency probes (20–100 MHz) allow for high-resolution imaging of nerve structures, enhancing detection of pathological states such as nerve enlargement at the site of compression, signs of impingement, nerve mobility, and vascularity [3,4]. Furthermore, by studying peripheral nerve infrastructures, one can identify different pathological states, such as the presence of tumors, nerve severance, and demyelinating processes [5,6]. A common clinical application is the use of ultrasound to assess compression of the median nerve, which is the pathophysiological cause of Carpal Tunnel Syndrome (CTS).

CTS is one of the most common problems that neurologists see in outpatient settings, accounting for about 90% of the referrals in neuromuscular clinics [7]. The pathophysiology of CTS is a result of compression of median nerve at the level of the wrist within the carpal tunnel. This leads to localized swelling of the nerve at the wrist while the forearm segment of the nerve remains the normal size. In mild cases, patients can present with sensory loss and pain in their hands. In severe cases, patients may present with muscle wasting and weakness, resulting in loss of motor function in the hand [8]. CTS is easily treatable using a wrist splint in mild cases and may need surgery in more severe cases. What is important is early identification of median nerve entrapment at the carpal tunnel and rule out other potential causes of hand symptoms before the median nerve damage is severe [9]. As such, electrodiagnostic tools such as nerve conduction studies and electromyography have traditionally been used in outpatient settings to assist with the diagnosis of CTS [10,11]. Imaging studies such as magnetic resonance imaging (MRI), computed tomography (CT) scans, and X-rays (XR) have also been used. More recently, clinicians have used ultrasonography (US) to identify localized median nerve pathology in the carpal tunnel [12].

There is a growing interest in employing ultrasound imaging for large-scale and longitudinal clinical studies to obtain reproducible nerve images [13]. However, the use of ultrasound for identifying anatomical structures presents challenges for those without proper training and expertise in ultrasound imaging analysis, especially with large amounts of data. To address these limitations, studies employing deep learning (DL) methods successfully identified peripheral nerves, including the median nerve. Hafiane et al. (2017) performed segmentation studies of the median nerve at the forearm, achieving an average Dice score of 0.850 [14]. Festen et al. (2021) implemented a U-Net using 5560 dynamic ultrasound images of the carpal tunnel region from 99 patients, where the median nerve was segmented, yielding averaged Dice scores of 0.77, 0.86, and 0.82 for finger, wrist, and both flexion movements, respectively [15]. Cosmo et al. (2021) implemented Mask R-CNN to perform median nerve segmentation from 151 US images obtained from 53 subjects with CTS, where a mean Dice coefficient score of 0.931 (0.883–0.967 range) was obtained from this study [16]. Smerelli et al. (2022) implemented Mask R-CNN to segment the median nerve on 246 US images obtained from 103 patients and obtained an average Dice score of 0.88 ± 0.19 [17]. Peng et al. (2024) developed an automated CTS diagnostic system that implemented a variant of the SegFormer network on 32,301 US images obtained from 130 videos from a total of 81 participants, making it one of the largest studies for CTS diagnosis and median nerve segmentation to date, achieving an average dice score of 0.8578 [18]. A recent study from Moser et al. (2024) trained a U-Net–based model on 2355 images covering the distal half of the forearm in 25 CTS patients and 26 healthy controls, achieving an average Dice score of 0.76 and demonstrating the feasibility of automated cross-sectional area measurement along the nerve course [19].

Building on prior work, our study contributes: (1) adaptation of a deep-learning segmentation model—previously validated for extraocular muscles on MRI to the median nerve; (2) evaluation at two clinically significant anatomical sites, the wrist and distal forearm–because the median-nerve cross-sectional area (CSA) wrist-to-forearm ratio exhibits the highest sensitivity for CTS assessment by ultrasound [20,21,22]; and (3) incorporation of datasets with varying nerve depths and ultrasound image qualities, alongside systematic augmentations to further test the model, increase the dataset and enhance generalizability. Collectively, these advances support the use of automated ultrasound segmentation as a practical adjunct to electrodiagnostic testing and pave the way for studies in patients with carpal tunnel syndrome (CTS) and for 3D nerve modeling.

## 2. Materials and Methods

### 2.1. Study Population

Healthy male and female volunteers, aged 18 and above, were recruited under the protocol “Investigational Use of Neuromuscular Ultrasound” (NCT 05237973) approved by the National Institutes of Health Institutional Review Board. Written consent was obtained from all subjects. The volunteers were in good general health, as evidenced by their medical history and physical examination. Medical conditions under control, such as hypertension, were acceptable. People with a history of strokes, muscle disorders, peripheral neuropathies, or spinal surgeries were excluded from the study. Hand and foot dominance was recorded. A neurological exam with attention to the presence of muscle weakness, sensory loss, or deep tendon reflex abnormalities was performed. Nerve conduction studies were performed using a Natus EMG/NCS device (Natus Medical Incorporated, Middleton, WI, USA) of the right median, ulnar, fibular, tibial motor nerves, and sural sensory nerves, using standard techniques.

### 2.2. Nerve Ultrasound

**Figure 1:**
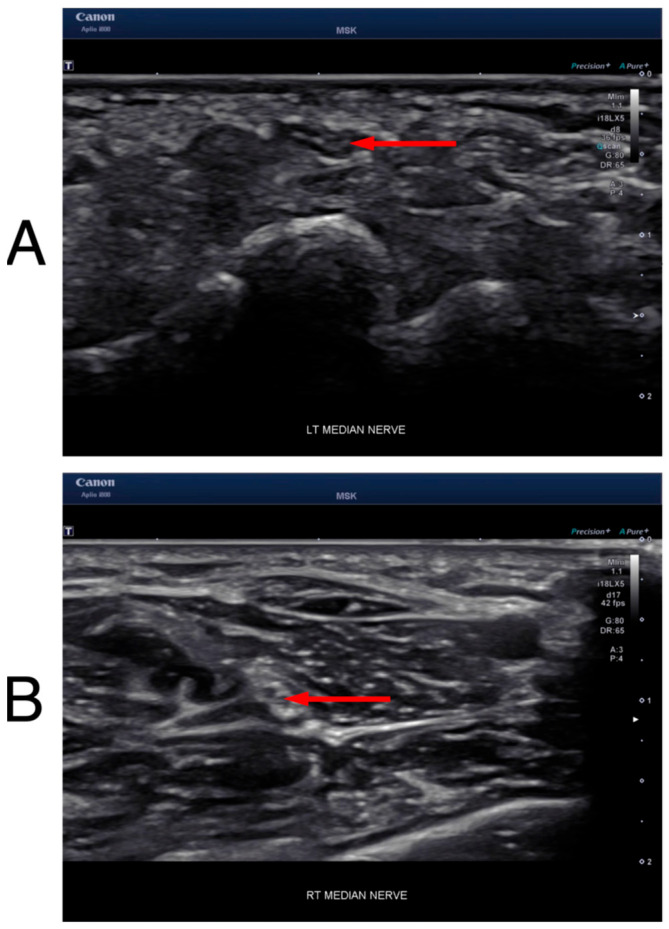
Sample frame from ultrasound video taken from (**A**) wrist crease and (**B**) distal forearm. The location of the median nerve is denoted by the red arrow.

Nerve US was performed using a Canon i800 ultrasound device (Canon Medical Systems USA, Inc., Tustin, CA, USA) with an 18 MHz linear probe (i18LX5). The subjects were placed in a supine position, with their arms supinated and wrists in a relaxed, neutral position. Static images of the median nerve were obtained to measure the CSA at the wrist crease and distal forearm. The dynamic imaging used in this study was performed by one neurologist, K.T., by obtaining videos of the nerve. The probe was placed transversely at the wrist crease to evaluate the median nerve at the wrist crease. Video clips were taken as the probe moved 1 cm back and forth along the longitudinal axis of the nerve to create image variability. Similarly, a second set of video clips was taken at the distal forearm, proximal to the pronator quadratus and in between the flexor digitorum superficialis (FDS) and flexor digitorum profundus (FDP), again moving back and forth longitudinally along the nerve over a 1 cm distance. Sample frames from the ultrasound of the wrist and forearm can be visualized in Figure 1.

### 2.3. Data Preprocessing

Prior to the dataset pre-processing, all ultrasound was anonymized to ensure participant confidentiality in accordance with the NIH IRB-approved protocol and informed consent. Each US video clip was assigned an alphanumeric code with no link to subject identifiers or specific demographic metadata.

**Figure 2:**
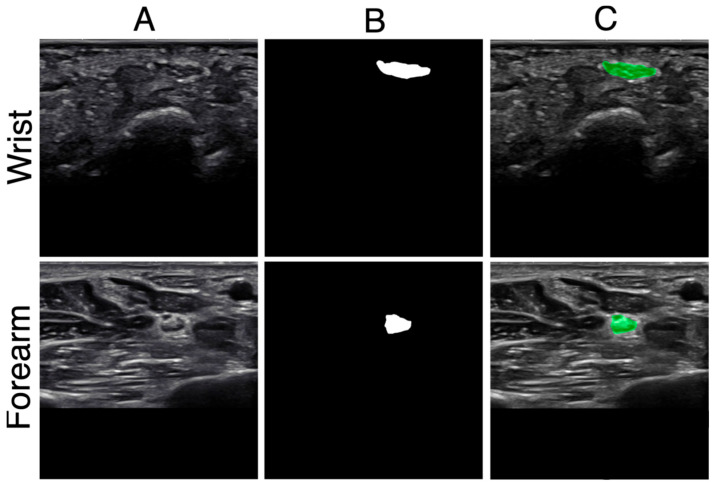
Representative images from the distal wrist and forearm: (**A**) Raw ultrasound image padded to 1024 × 1024; (**B**) expert-traced ground truth mask padded to the same dimensions; (**C**) ground truth mask (green) overlaid on the padded raw image.

For the first dataset, video clips were converted into individual frames, and two datasets of 500 images each were acquired and curated from 8 healthy volunteers: one at the wrist crease and the other at the distal forearm, located between the FDS and FDP, proximal to the pronator quadratus. As the images from the dataset were obtained from Tdeidentified data, the number of images contributed by each volunteer could not be tracked. For the second dataset, an additional 35 wrist and 26 forearm still images were acquired from the healthy volunteers to increase variability. A neurologist manually annotated (KT) the median nerve images using Fiji to obtain a pixel-based trace of the median nerve, which was used as ground truth for the DL model. All images were first cropped to 1024 × 690 pixels. Then, using MATLAB version R2023b, the tracings were filled to generate binary masks. Finally, padding was added to resize the image to 1024 × 1024 for input into the DL model. Figure 2 illustrates representative wrist and forearm images, including the padded raw ultrasound, the corresponding expert-traced ground truth mask, and the mask overlaid on the raw image.

**Figure 3:**
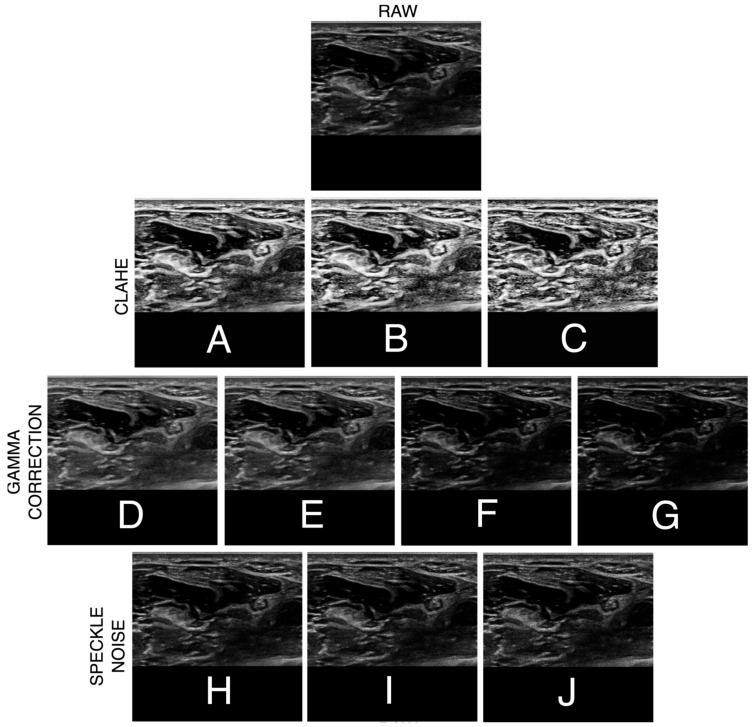
Sample representation of the individual augmentations performed on a US image obtained at the distal forearm (top image) from the second dataset. First row displays augmentation using contrast-limited adaptive histogram equalization (CLAHE) with (**A**) Clip-Limit of 0.01 with 8 × 8 tile size, (**B**) Clip-Limit of 0.02 with 8 × 8 tile size, and (**C**) Clip-Limit of 0.03 with 16 × 16 tile size. The second row shows gamma correction with (**D**) γ = 0.70, (**E**) γ = 0.80, (**F**) γ = 1.20, and (**G**) γ = 1.30. Finally, the third row shows the addition of speckle noise at (**H**) 0.10, (**I**) 0.20, and (**J**) 0.30.

The first dataset (500 wrist and 500 forearm images) was used without augmentation to establish baseline model performance. The second dataset (35 wrist and 26 forearm images) underwent intensity-based augmentation to enhance robustness across variable imaging conditions. Each original image was retained as a reference and expanded into ten augmented variants, yielding 385 (35 × 11) wrist and 286 (26 × 11) forearm image-mask pairs. Contrast Limited Adaptive Histogram Equalization (CLAHE) was introduced with three parameter settings (Clip-Limit of 0.01 with 8 × 8 tile size, Clip-Limit of 0.02 with 8 × 8 tile size, and Clip-Limit of 0.03 with 16 × 16 tile size) [23]. Gamma correction with four values was also applied (γ = 0.70, 0.80, 1.20, 1.30) [24]. Finally, speckle noise was added at three levels (0.10, 0.20, 0.30) [23]. Each augmentation type (CLAHE, gamma correction, and speckle noise) with various parameters was applied independently to the originalstill image rather than in combination. Each parameter was chosen based on pilot visual assessment to mimic variations in gain, exposure, and speckle that can arise from differences in ultrasound probe settings and tissue depth during acquisition. Examples of each individual augmentation are shown in Figure 3. Combining the first and second dataset (with augmentations) resulted in 885 (500 + 385) wrist and 786 (500 + 286) forearm image–mask pairs, which were used to train and evaluate the deep learning model for sensitivity to acquisition variability and noise.

### 2.4. Deep Learning Model

The deep learning model implemented in this study is the U-Net, a type of encoder–decoder-based convolutional neural network (CNN), initially developed by Ronneberger (2015) and commonly used in biomedical image segmentation [25]. We utilized a 4-layered U-Net model, previously employed for extraocular muscle segmentation, and applied it to the segmentation of the median nerve [26]. The model was implemented in TensorFlow/Keras and trained on a Lambda Vector workstation equipped with an Intel Core i9-10980XE 3.00 GHz CPU and dual NVIDIA RTX A5000 GPU. Rectified linear unit (ReLU) activation functions were used in the encoder, with SoftMax applied at the final layer. Training employed categorical cross-entropy loss with the Adam optimizer (learning rate = 0.0001). Training was conducted for 100 epochs with a batch size of four. These hyperparameters were selected based on our prior experience with medical segmentation tasks and GPU memory limitations. No formal ablation study or early-stopping strategy was implemented, rather the model was trained for the full 100 epochs to observe the convergence behavior under a consistent learning rate and batch value. Once trained, the model was evaluated on the test data, and the predicted segmentation masks were subjected to quantitative performance analysis as described in Section 2.5.

### 2.5. Deep Learning Model Performance Analysis

Segmentation performance was quantified using the Dice coefficient and Intersection-over-Union (IoU), which measure the pixel-wise overlap between predicted and ground-truth masks. Both metrics range from 0 to 1, with a score of 0 indicating no overlap and a score of 1 indicating perfect overlap. Dice and IoU are defined in Equations (1) and (2). In these equations, true positives (*TP*) are correctly predicted nerve pixels, true negatives (*TN*) are correctly excluded background pixels, false positives (*FP*) are background pixels incorrectly classified as nerve, and false negatives (*FN*) are nerve pixels missed by the model.

(1) $$Dice=\frac{2\times TP}{\left(TP+FP)+(TP+FN\right)}$$

(2) $$IoU=\frac{TP}{TP+FP+FN}$$

To provide a more comprehensive assessment, additional metrics were calculated, including accuracy (Equation (3)), precision (Equation (4)), specificity (Equation (5)), and sensitivity (Equation (6)) [27].

(3) $$Accuracy=\frac{TP+TN}{TP+TN+FP+FN}$$

(4) $$Precision=\frac{TP}{TP+FP}$$

(5) $$Specificity=\frac{TN}{TN+FP}$$

(6) $$Sensitivity=\frac{TP}{TP+FN}$$

Finally, cross-sectional area (CSA) agreement between manual and automated segmentation was evaluated using test images from first, second and combined datasets. The multiple testing values from each subject were treated as independent entities. Predicted masks from the test set were analyzed in MATLAB alongside manually traced masks. The agreement was examined using intraclass correlation coefficients (ICC (2,1), two-way mixed model, single rater), Pearson correlation, and Bland–Altman analysis. Together, these measures assessed both pixel-level segmentation accuracy and the reliability of CSA estimates for clinical application.

## 3. Results

### 3.1. Demographics

Median nerve ultrasound images at the distal forearm and the wrist were obtained from 37 healthy volunteers. For the first dataset, video clips from 8 individuals from this population were used for analysis. For the second dataset, only 35 still images from the wrist and 26 still images from the forearm were available from the original cohort of 37 patients due to suboptimal image quality. Nerve conduction studies confirmed that the median nerve was neurophysiologically normal, and nerve US did not detect median nerve enlargement, as noted in Table 1. Since all images from the subjects were deidentified, as per research agreement, we cannot distinguish the demographic information between first and second dataset.

### 3.2. Results from the First Dataset

For the first dataset (500 image-mask pairs per anatomical site: forearm and wrist) obtained from video clips, the model was trained on 400 images (80%) and tested on 100 images (20%). Training the model took approximately 30 min. Segmentation performance for the test sets is summarized in Table 2. At the forearm, the U-Net achieved a mean IoU of 0.844 (±0.077) and a Dice score of 0.913 (±0.053). At the wrist, performance was slightly higher, with a mean IoU of 0.876 (±0.047) and a Dice score of 0.933 (±0.030). Accuracy, precision, sensitivity, and specificity were all high at both sites, indicating robust segmentation.

**Figure 4:**
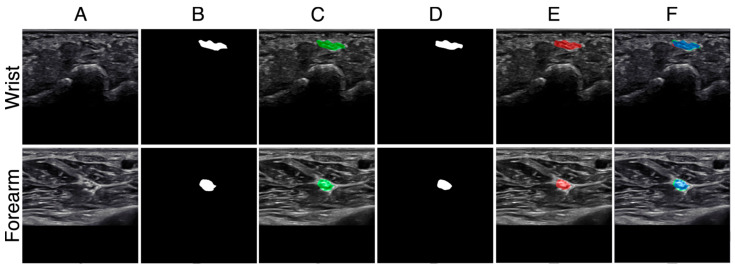
Examples shown for the wrist crease (**top row**) and distal forearm (**bottom row**). (**A**) Raw padded ultrasound image; (**B**) expert-traced ground truth (GT) mask; (**C**) GT mask (green) overlaid on the raw image; (**D**) U-Net predicted mask; (**E**) predicted mask (red) overlaid on the raw image; (**F**) combined GT (green) and prediction (red), with overlap shown in blue. For the illustrated cases, the wrist segmentation achieved IoU = 0.873 and Dice = 0.932, while the forearm segmentation achieved IoU = 0.779 and Dice = 0.876.

Representative test examples are shown in Figure 4, including raw ultrasound frames, expert ground-truth masks, U-Net predictions, and corresponding overlays. In the examples, the wrist segmentation achieved IoU of 0.873 and Dice of 0.932, while the forearm segmentation achieved IoU of 0.779 and Dice of 0.876. These results served as the baseline against which all subsequent augmentation and generalization experiments were compared.

### 3.3. Results from the Second Dataset

To evaluate the generalizability of the baseline models, we applied Section 3.2 models of the wrist and forearm to 35 newly acquired still images from the wrist and 26 from the forearm. A marked performance decline was observed (forearm IoU of 0.185, Dice of 0.254; wrist IoU of 0.137 and Dice of 0.188), which revealed sensitivity to variations in nerve depth, tissue morphology, and ultrasound intensity. This result underscored the need for additional image diversity to improve model robustness.

To address this, each image from the second dataset (the new 35 still wrist images and 26 still forearm images) underwent three categories of intensity-based augmentation (CLAHE, gamma correction, speckle noise) producing 10 augmented variants per image (Section 2.3). We then trained models exclusively on the new augmented dataset (N = 385 wrist still images; N = 286 forearm still images) and tested it on the first baseline dataset. This initial validation improved generalization substantially yielding forearm IoU of 0.408 and Dice of 0.533, and wrist IoU of 0.705 and Dice of 0.820.

Subsequently, the augmented dataset was evaluated internally under an 80–20 train–test split (308 training/77 testing wrist images; 229 training/57 testing forearm images). This yielded strong within-the-set performance forearm IoU of 0.944 and Dice 0.971, wrist IoU of 0.951 and Dice 0.974.

### 3.4. Results from the Combined First and Second Datasets

To evaluate the applicability of our methods, both datasets (baseline and new augmented dataset) were merged, yielding 885 wrist and 786 forearm image–mask pairs to assess the integrated performance of mixed-dataset-enhanced models.

The combined dataset underwent five trials, each with a randomized 80–20 train–test split, producing N = 177 testing images from the wrist and N = 157 testing images from the forearm, to ensure consistency with the model and demonstrate that, despite added variations in data where the nerve may be at different depths or have varying image quality, the model is still able to locate the nerve.

**Figure 5:**
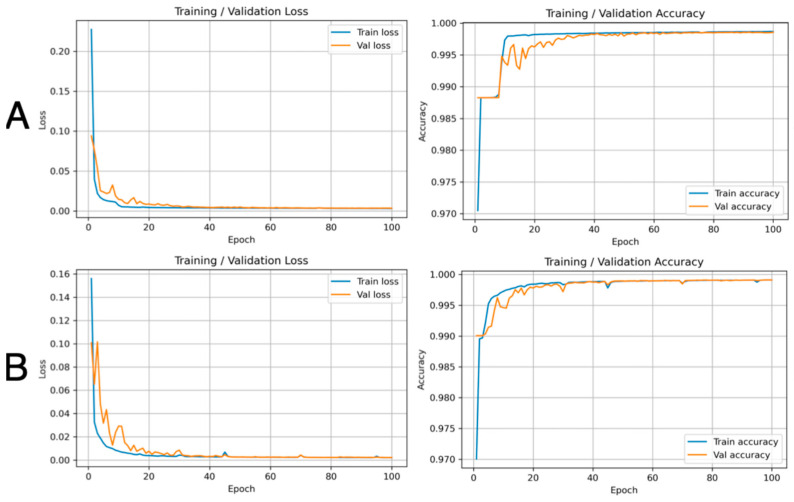
Representative Training and Validation learning curve for wrist-based median nerve segmentation. Row (**A**): Baseline dataset. Row (**B**): Combined dataset (baseline and augmented second dataset).

**Figure 6:**
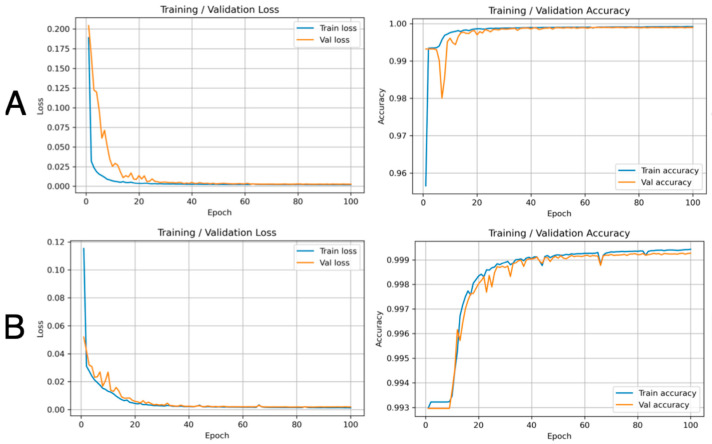
Representative Training and Validation learning curves for forearm-based median nerve segmentation. Row (**A**): Baseline dataset. Row (**B**): Combined dataset (baseline and augmented second dataset).

**Figure 7:**
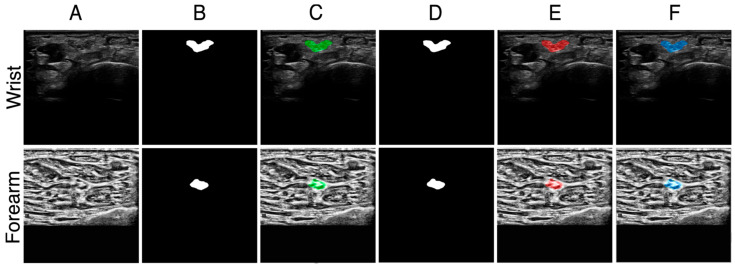
Sample representation of the results obtained from the combined batch of the data. Testing data shown for both the forearm (augmented with CLAHE with Clip-Limit of 0.03 with tile-size of 16 × 16) and wrist (augmented with speckle noise addition at 0.30). (**A**) Raw padded US image; (**B**) Ground truth mask; (**C**) Ground truth (green) overlayed on the raw US image; (**D**) U-Net predicted mask; (**E**) U-Net predicted mask (red) overlayed on the raw US image; (**F**) Both the ground truth (green) and U-Net predicted mask (red) overlayed on top of each other on the raw US image, where the overlap is shown in blue. For the sample representation shown for the wrist, the IoU was 0.970 with a Dice of 0.985. For the forearm, the IoU was 0.946 with a Dice of 0.972.

**Figure A1:**
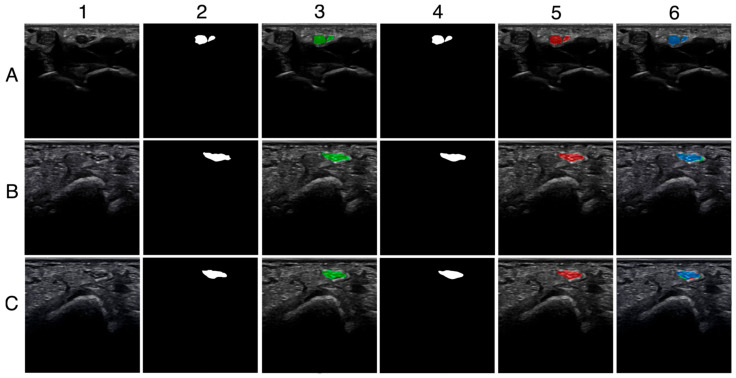
Representative segmentation results for the wrist (from the combined data batch). (1) raw US image, (2) ground truth (GT) mask, (3) GT mask (green) overlaid on the raw image, (4) U-Net predicted mask, (5) U-Net prediction (red) overlaid on the raw image, and (6) combined GT (green) and prediction (red) overlay on the raw image where the overlap is shown in blue. Rows correspond to individual cases: (**A**) A case in which IoU is 0.965 and Dice is 0.982, (**B**) A case where IoU is 0.889 and Dice is 0.941, and (**C**) A case where the results are poor at IoU being 0.832 and Dice at 0.909.

**Figure A2:**
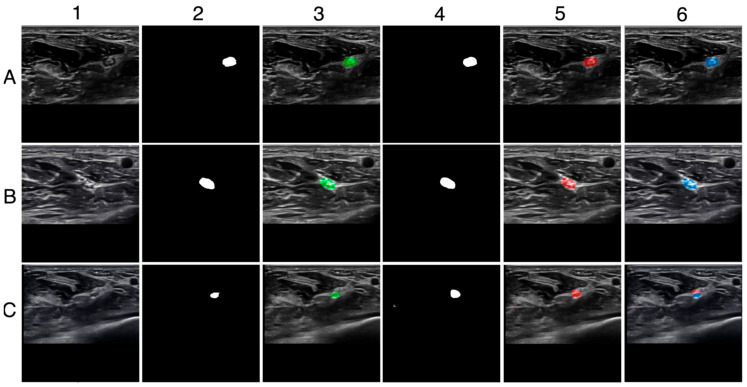
Representative segmentation results for the forearm (from the combined data batch). (1) raw US image, (2) ground truth (GT) mask, (3) GT mask (green) overlaid on the raw image, (4) U-Net predicted mask, (5) U-Net prediction (red) overlaid on the raw image, and (6) combined GT (green) and prediction (red) overlay on the raw image where the overlap is shown in blue. Rows correspond to individual cases: (**A**) A case in which IoU is 0.966, Dice is 0.983, (**B**) A case where IoU is 0.928 and Dice is 0.963, and (**C**) A case where the results are poor at IoU being 0.537 and Dice at 0.698.

Prior to quantitative evaluation, we examined the learning curves for models trained on the baseline versus combined datasets to visualize convergence behavior and potential overfitting. For both the wrist (Figure 5) and forearm (Figure 6), the baseline models (Row A) showed rapid decreases in training loss and increases in accuracy, but with a modest separation between training and validation loss and accuracy during approximately the first 10–20 epochs, most notably for the forearm. In contrast, when the model was trained on the combined dataset that included the augmented images (Row B), the validation curves became smoother, exhibited fewer early oscillations, and more closely tracked the corresponding training curves throughout the 100 epochs. The final training and validation losses and accuracies were nearly identical in all settings, indicating no pronounced overfitting; however, the reduced train–validation divergence and variability with the combined dataset are consistent with a regularizing effect of augmentation and the improved IoU/Dice performance summarized in Table 3. Representative visualizations of the wrist and forearm from the combined dataset are shown in Figure 7, Appendix A Figure A1 and Figure A2. At the wrist, IoU values range from 0.917 to 0.924, with Dice coefficients of 0.956 to 0.960. At the forearm, the mean IoU across trials ranged from 0.882 to 0.895, with a corresponding Dice coefficient of 0.935 to 0.943. These improvements were consistent across all trials, demonstrating enhanced robustness of the U-Net mode with augmented data.

### 3.5. CSA Reliability Analysis

**Figure 8:**
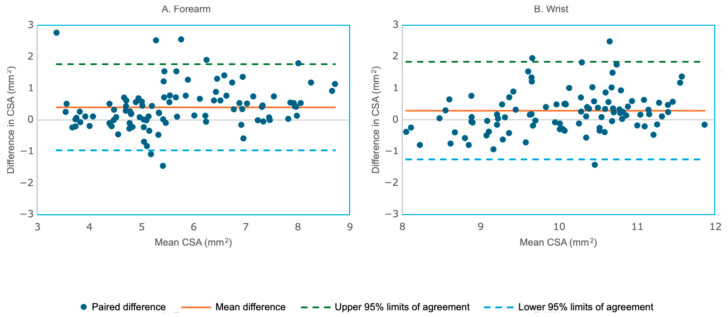
Bland–Altman plot comparing the manual and DL method measurements of the forearm cross-sectional area (CSA) from the first dataset (one outlier with a difference of 4.6 mm^2^ was not shown in the wrist plot). (**A**). Forearm (N = 100) showed a mean paired difference was 0.402 ± 0.696, indicating a significant bias. (**B**). Wrist (N = 100) showed a mean paired difference of 0.294 ± 0.79, also indicating a significant systematic bias.

In the first dataset from Section 3.2, the forearm data (N = 100 testing images) demonstrated higher manual CSA estimates (mean ± SD: 5.92 ± 1.46) compared to DL (5.52 ± 1.31). The mean paired difference was 0.402 ± 0.696, which was statistically significant (t = 5.77, *p* < 0.0001; 95% CI: 0.264–0.540), indicating a systematic bias. Strong agreement was observed with Pearson r = 0.879 (95% CI: 0.825–0.917, *p* < 0.001) and ICC (2,1) = 0.874 (95% CI: 0.818–0.913), indicating a high degree of reliability. The Bland–Altman plot of the forearm (Figure 8A) further illustrated this bias, with the mean difference line positioned above zero, while the narrow limits of agreement reflected the strong consistency between methods. At the wrist (N = 100 testing images), manual CSA (10.22 ± 1.03) also exceeded DL (9.92 ± 0.95), yielding a mean difference of 0.294 ± 0.79 that was significant bias (t = 3.73, *p* < 0.001; 95% CI: 0.138–0.451). Correlation and reliability were lower than at the forearm but remained acceptable (r = 0.684, 95% CI: 0.564–0.776, *p* < 0.001; ICC (2,1) = 0.682, 95% CI: 0.561–0.774. Figure 8B shows the Bland–Altman plot comparing the manual and DL model-based segmentations of the wrist CSA dataset, illustrating the pattern.

**Figure 9:**
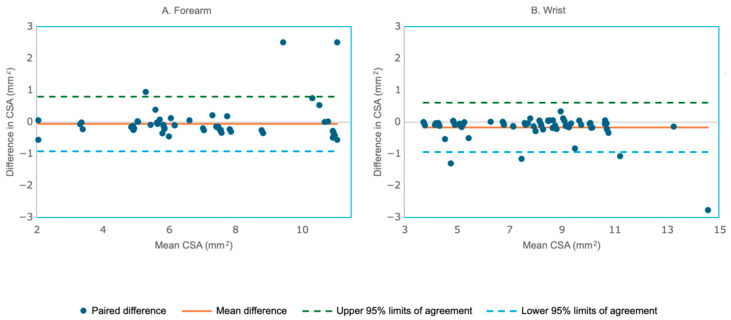
Bland–Altman plot comparing the manual and DL method measurements of the forearm cross-sectional area (CSA) from the second dataset. (**A**). Forearm (N = 57) showed a mean paired difference of −0.056 ± 0.438 indicating no systematic bias. (**B**). Wrist (N = 77) showed a mean paired difference of −0.168 ± 0.397, indicating a significant systematic bias.

In the second dataset from Section 3.3, the forearm data (N = 57 testing images) demonstrated similar manual CSA estimates (mean ± SD: 6.93± 2.30) compared to DL (6.98 ± 2.28). The mean paired difference was −0.056 ± 0.438, which was not statistically significant (t = 0.96, *p* = 0.3402; 95% CI: −0.172–0.061), indicating no systematic bias. Strong agreement was observed with Pearson r = 0.982 (95% CI: 0.969–0.989) and ICC (2,1) = 0.982 (95% CI: 0.969–0.989), indicating a high degree of reliability. The Bland–Altman plot (Figure 9A) further showed the narrow limits of agreement reflecting the strong consistency between methods. At the wrist (N = 77 testing images), demonstrated higher manual CSA estimates (mean ± SD: 7.74 ± 2.33) compared to DL (7.91 ± 2.44). The mean paired difference was −0.168 ± 0.397, that was significant (t = 3.71, *p* = 0.0004; 95% CI: −0.258–−0.078), indicating a slight systematic bias. Correlation and reliability were similar as those at the forearm (r = 0.987, 95% CI: 0.980–0.992; ICC (2,1) = 0.984, 95% CI: 0.970–0.990). Figure 9B shows the Bland–Altman plot comparing the manual and DL model segmentations of the wrist CSA from the second dataset.

**Figure 10:**
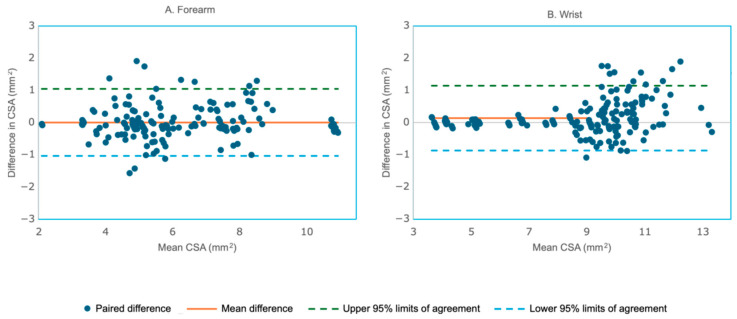
Bland–Altman plot comparing the manual and DL method measurements of the forearm cross-sectional area (CSA) from the combined dataset. (**A**). Forearm (N = 157) showed a mean paired difference of 0.008 ± 0.53, indicating no systematic bias. (**B**). Wrist (N = 177) showed a mean paired difference of 0.138 ± 0.51, indicating a significant systematic bias.

In the combined dataset from Section 3.4 the agreement between manual and DL measurements improved significantly. For the forearm (N = 157 testing images), manual CSA (6.13 ± 1.95) and DL CSA (6.12 ± 1.95) were virtually identical. The mean difference was 0.008 ± 0.53, which was not significant (t = 0.190, *p* = 0.850; 95% CI: −0.076 to 0.091). The reliability between the two methods was excellent, with r = 0.964 (95% CI: 0.951–0.974, ICC (2,1) = 0.964 (95% CI: 0.951–0.973). Figure 10A shows the Bland–Altman plot comparing the manual and DL model-based segmentation of the forearm CSA from the combined data. In the combined dataset, the wrist (N = 177 testing images), manual CSA (8.64 ± 2.52), and DL CSA (8.50 ± 1.79) had a mean difference of 0.138 ± 0.51, a small but statistically significant offset (t = 3.59, *p* = 0.0004; 95% CI: 0.062–0.215), indicating a systematic bias. Correlation and reliability were near-perfect with r = 0.980 (95% CI: 0.973–0.985, *p* < 0.00001), ICC (2,1) = 0.978 (95% CI: 0.971–0.984). Figure 10B shows the Bland–Altman plot comparing the manual and DL model-based segmentation of the wrist CSA from the combined data.

Overall, combining the first and second datasets eliminated the forearm bias seen in the first dataset and substantially improved the wrist agreement, leaving a minor residual offset that should be acknowledged in future analyses.

## 4. Discussion

This study demonstrates that DL models such as U-Net can accurately identify the median nerve at both the superficial level of the wrist and the deeper tissues of the distal forearm. Importantly, segmentation at these two anatomically distinct sites was achieved with high certainty and reproducibility using a single U-Net.

Prior studies reported Dice scores for median nerve segmentation have ranged from 0.76 to 0.93, depending on anatomical site, model architecture, and task complexity, as noted in Table 3. Traditional non-DL-based approaches, such as edge/phase information with active contours, reached a Dice score of 0.85 for the forearm [14]. More recent CNN-based methods, including Mask R-CNN and U-Net at the wrist, reported Dice scores of 0.77 to 0.93 [15,16,17]. With a transformer backbone (SegFormer-B2), Mask R-CNN achieved a Dice score of 0.86 for wrist-based segmentation [18]. At the distal forearm, Moser et al. (2024) reported a Dice score of 0.76 using U-Net [19]. 

In comparison, our U-Net results are favorable for both the forearm and wrist. In the first dataset, Dice scores were 0.91 (forearm) and 0.93 (wrist). After augmentation, the performance of the combined dataset improved to 0.94 (forearm) and 0.96 (wrist). Notably, our wrist results outperform those of prior transformer- and Mask R-CNN-based methods (Table 4), while our forearm Dice score is substantially higher than previously published values. Differences across studies may reflect variations in patient cohorts, ultrasound scanners, and labelling protocols.

Beyond IoU and Dice metrics, CSA analysis provided further validation. In the first dataset, we detected a significant manual–DL bias at both sites (forearm: mean Δ = 0.402, d = 0.577; wrist: Δ = 0.294, d = 0.373), despite strong to very strong correlations and high ICCs. After augmentation (combined dataset), the forearm bias was effectively eliminated (Δ = 0.008, *p* = 0.850, d = 0.015), with r = 0.964 and ICC (single) = 0.964, indicating excellent concordance. At the wrist, augmentation substantially improved association (r = 0.980) and reliability (ICC (2,1) = 0.978), while reducing—but not fully removing—a small positive manual–DL offset (Δ = 0.138; t = 3.59, *p* = 0.0004; d = 0.514). This offset is approximately 1–2% of the diagnostic thresholds for median nerve CSA at the wrist, with an average of 9.8 mm^2^ (STD of 2.4 mm^2^) [28]. The magnitude of this difference is smaller than the level of manual tracing precision and would not alter clinical interpretation. Together, these findings suggest that augmentation strategies addressing intensity and texture variability can further enhance segmentation accuracy and CSA estimation, and that a 4-layer U-Net can generalize effectively across two anatomically and acoustically distinct sites (distal forearm vs. wrist).

Contextualizing our findings within the literature, our Dice values align with and often exceed the upper range of previously reported results for the wrist and show clear improvement at the forearm. This trend is consistent with a recent meta-analysis by Wang (2023), which pooled results from diverse architectures (Mask R-CNN, generic CNNs, U-Net) and reported a mean Dice of approximately 0.90 for wrist segmentation, placing our wrist performance within this benchmark [29]. Nevertheless, cross-study comparisons must be interpreted cautiously, as differences in scanner settings, probe frequency, dynamic maneuvers (e.g., finger vs. wrist flexion), subject pathology (CTS vs. healthy), and labeling protocols can significantly affect performance, as illustrated by Festen et al. (2021), who reported task-dependent Dice scores ranging from 0.77 to 0.86 [15]. Finally, our CSA analyses highlight a subtle, yet reproducible wrist offset after augmentation, which may reflect anatomical or positional variability as well as speckle statistics at the wrist. 

Practically, the combination of high Dice and IoU scores, strong Pearson correlations, and excellent ICCs—particularly the absence of meaningful bias at the forearm—supports the clinical utility of automated CSA estimation in routine ultrasound scanning. At the wrist, the small residual bias is unlikely to be clinically consequential in longitudinal monitoring; however, it should be acknowledged when applying threshold-based decision support.

A limitation of the study is that due to the data anonymization and the randomized train–test split, ultrasound frames from the same subject may have appeared in the experiments. While this approach ensured participant confidentiality and compliance with the IRB protocols, there is the concern of introducing limited intra-subject similarities between the training and testing sets. Further work should extend validation to cohorts with CTS patients, perform subject-specific segmentation, explore additional augmentation techniques, DL-model hyperparameter ablation studies and early-stopping, and incorporate analyses of inter-rater variability. Consistent with the quantitative improvements observed with the augmented dataset, the training and validation learning curves (Figure 5 and Figure 6) demonstrate smoother convergence, reduced early-epoch fluctuations, and closer alignment between training and validation performance, supporting the role of augmentation in improving training stability and generalization. Moreover, applying object tracking algorithms such as YOLO-Nas in combination with DL methods on ultrasound video could enable dynamic nerve segmentation, as opposed to the static analysis in our study [30]. One benefit of performing dynamic nerve segmentation is to study the mobility of the median nerve at the wrist during dynamic finger and wrist movements. There are studies that show reduced gliding of the median nerve, correlating with the severity of CTS [21]. Furthermore, applying 3D segmentation to obtain volumetric analysis of the median nerve may enhance the visualization of fascicular morphology changes in CTS and provide additional metrics in assessing CTS severity. There are emerging studies that show pathological changes of the nerve fascicles with different neuropathic states, such as chronic inflammatory demyelinating polyneuropathy and Charcot Maie Tooth disease [31].

In addition to CNN based on encoder–decoder architectures such as U-Net, transformer-based and hybrid deep learning models have demonstrated potential for medical image interpretation. Models such as ViT-AMCNet (fusion of attention mechanism-integrated convolution, AMC, with the vision transformer, ViT tested on laryngeal tumors), MamlFormer (transformer network with manifold adversarial multi-model learning tested on laryngeal tumors), CGAM (causality graph attention mamba network tested on ESCC), FDT (feature disentangled transformer tested on SCC) have been used to achieve good interpretability and grading performance of tumors in histopathological slides [32,33,34,35]. In terms of classification, the Swin Transformer and Focal-Loss-Swin-Transformer Networks have been recently used to achieve an average classification accuracy of at least 85% lung adenocarcinoma subtype classification [36,37]. Integrating such frameworks into ultrasound-based anatomical region segmentations could further enhance feature representation and cross-domain generalization.

Deep learning approaches have been applied beyond the biomedical field in non-destructive ultrasonic evaluation tasks such as concrete damage assessment and weld-defect detection, where the models learn acoustic and structural patterns from speckle-rich ultrasonic signals to produce meaningful outputs for evaluation [38,39]. Such analysis aligns with quantitative ultrasound (QUS) techniques that analyze echo intensity, scatter properties, and spectral features of regions of interest [40]. As a further approach in the ultrasound-based analysis of the median nerve, QUS-derived biomarkers coupled with DL-based segmentation may enhance the interpretation of the nerve echotexture, improve robustness across scanning conditions, and enable extraction of quantitative biomarkers beyond the CSA.

As these various approaches become increasingly efficient and innovative, their adaptations represent a promising direction for further research for potential applications towards clinical assessment, surgical planning, and biomechanical modelling.

## Figures and Tables

**Table 1:** Patient demographics.

Characteristics (Total N = 37)	Findings
Age—median (range)	39 (21–77)
Sex	21F (57%)
Right Handedness	33 (89%)
**Electrophysiology (mean ± STD)**	
Median Motor Nerve (Amplitude/distal latency/conduction velocity)	11.7 ± 3.2 mV/3.2 ± 0.4 ms/59.1 ± 4.7 m/s
Median Sensory Nerve (Amplitude/conduction velocity)	43.3 ± 17.5 µV/57.5 ± 6.1 m/s

Abbreviations: mV—millivolts, ms—milliseconds, m/s—meters/second, µV—microvolt, F—female, STD—standard deviation.

**Table 2:** Segmentation performance of the U-Net model on the test images from the first dataset (N = 100 forearm test images; N = 100 wrist test images). The total number of images in the first dataset was N = 500 forearm images and N = 500 wrist images.

Performance Metric	Forearm (N = 100)	Wrist (N = 100)
IoU ± STD	0.844 ± 0.077	0.876 ± 0.047
Dice ± STD	0.913 ± 0.053	0.933 ± 0.030
Accuracy	0.999	0.999
Precision	0.947	0.947
Specificity	1.000	0.999
Sensitivity	0.888	0.922

**Table 3:** Median nerve segmentation metrics obtained from the U-Net model for both the forearm and the wrist from the testing batch of the combined dataset. To test consistency, five trials (splits) were conducted, each with a consistent 80–20 train–test split.

Anatomical Location (Number of Testing Images)	Average Metric ± STD	Split 1	Split 2	Split 3	Split 4	Split 5
Wrist (N = 177)	IoU ± STD	0.922 ± 0.048	0.924 ± 0.038	0.917 ± 0.047	0.918 ± 0.045	0.917 ± 0.044
	Dice ± STD	0.956 ± 0.027	0.960 ± 0.021	0.956 ± 0.027	0.956 ± 0.025	0.956 ± 0.024
Forearm (N = 157)	IoU ± STD	0.895 ± 0.075	0.892 ± 0.071	0.882 ± 0.076	0.892 ± 0.073	0.892 ± 0.082
	Dice ± STD	0.943 ± 0.044	0.942 ± 0.042	0.935 ± 0.046	0.941 ± 0.042	0.941 ± 0.055

**Table 4:** Comparison of prior studies on median nerve segmentation from ultrasound at the forearm, wrist, or both using non-DL or DL-based approaches.

*Study*	Model	Image Data	Results
*Hafiane (2017)* *[14]*	Probabilistic, edge phase information, and active contours	Average 500 US frames per patient (10 patients) obtained at the forearm	Dice: 0.85 (forearm)
*Festen (2021)* *[15]*	U-Net	5560 dynamic US images (99 patients) obtained at the wrist (carpal tunnel inlet)	Dice: 0.77 (finger flexion), 0.86 (wrist flexion), 0.82 (both flexion dataset)
*Cosmo (2021)* *[16]*	Mask R-CNN	151 US images (53 patients) from the wrist (carpal tunnel inlet)	Dice: 0.93 (wrist)
*Smerilli (2022)* *[17]*	Mask R-CNN	246 US images (103 patients) obtained at the wrist (carpal tunnel inlet)	Dice: 0.88 (wrist)
*Peng (2024)* *[18]*	OSA-CTSD method using Segformer B2 variant	32,301 US images (from 130 videos from 81 participants) obtained at the wrist	IoU: 0.76 (wrist) Dice: 0.86 (wrist)
*Moser (2024)* *[19]*	U-Net	2355 US images (25 CTS patients; 26 healthy) obtained at the distal forearm	Dice: 0.76 (distal forearm)
*OUR STUDY (2025)*	U-Net	First dataset: 500 forearm images and 500 wrist images from 8 patient videos Second dataset: 26 forearm images and 35 wrist images Totals: 786 forearm images, 885 wrist images (37 healthy patients)	IoU: 0.84 (forearm, first dataset), 0.89 (forearm, combined dataset), 0.88 (wrist, first batch), 0.92 (wrist, combined dataset) Dice: 0.91 (forearm, first dataset), 0.94 (forearm, combined dataset), 0.93 (wrist, first dataset), 0.96 (wrist, combined dataset)

## Data Availability

Data available from authors upon request.
